# Genome Sequence of Basidiomycetous Yeast Mrakia gelida MGH-2, Isolated from Skarvsnes Ice-Free Area, East Antarctica

**DOI:** 10.1128/mra.01064-22

**Published:** 2022-12-14

**Authors:** Masaharu Tsuji, Jun-ichi Ishihara, Atsushi Toyoda, Hiroki Takahashi, Sakae Kudoh

**Affiliations:** a Department of Materials Chemistry, National Institute of Technology (KOSEN), Asahikawa College, Asahikawa, Hokkaido, Japan; b Medical Mycology Research Center, Chiba University, Chiba, Chiba, Japan; c Advanced Genomics Center, National Institute of Genetics, Mishima, Shizuoka, Japan; d National Institute of Polar Research (NIPR), Tachikawa, Tokyo, Japan; e Department of Polar Science, The Graduate University for Advanced Studies, Tachikawa, Tokyo, Japan; Vanderbilt University

## Abstract

Basidiomycetous yeast Mrakia gelida MGH-2 has been reported from Surikogi Ike in the Skarvsnes ice-free area, East Antarctica. Here, we report on the high-quality genome sequence of the *Mrakia gelida* MGH-2 strain analyzed by PacBio Sequel and HiSeq 2500 instruments.

## ANNOUNCEMENT

Basidiomycetous yeast Mrakia gelida MGH-2 was isolated from lake sediment of Surikogi Ike in the Skarvsnes ice-free area, East Antarctica, by the 48th Japanese Antarctic Research Expedition (JARE-48) (1). This strain has high growth and alcohol fermentation abilities even at 4°C (2).

The MGH-2 strain culture and DNA extraction methods are the same as those we reported previously (3, 4). Briefly, yeast cells were cultured in yeast extract-peptone-dextrose (YPD) liquid medium (Difco) at 15°C for 5 days. The cells from cultures were collected by centrifugation at 3,500 × *g* for 5 min at 4°C. The cell pellets were washed with sterile distilled water and were precipitated by centrifugation again. The cell pellets were dried by a vacuum freeze dryer. Then, the lyophilized cells were powdered in a mortar and used for DNA extraction. The genomic DNA of MGH-2 was extracted and purified using a NucleoBond AXG100 column with a Nucleo buffer set III (TaKaRa Bio) according to the manufacturer’s protocol. Concentration and purification of the genomic DNA were determined by NanoDrop (Thermo Scientific) and the Qubit double-stranded DNA (dsDNA) broad-range (BR) assay kits (Thermo Scientific). Then, the genomic DNA was divided into two parts, namely, one for the PacBio Sequel platform and the other for the HiSeq 2500 platform (Illumina, CA) for use in sequencing. For the PacBio Sequel platform, a single-molecule real-time (SMRT) sequence library was constructed using a SMRTbell express template prep kit v1.0 (Pacific Bioscience, CA) according to the manufacturer’s protocol. Two sequencing libraries were size selected using the BluePippin system (Saga Science, MA) with a minimum fragment length cutoff of 20 kbp and 30 kbp. Each library was sequenced on one SMRT cell with binding kit 1.0/sequencing kit 1.2.1 and binding kit 2.0/sequencing kit 2.0, respectively. The run time for the movies was 360 min and 600 min, yielding a total of 1,133,939 polymerase reads. In this study, default parameters were used except where otherwise noted. In the PacBio Sequel sequencing, read quality control was performed prior to assembly by measuring the average length and concordance of spike-in controls and the distribution of read lengths in the sample. PacBio reads were assembled using the FALCON-Unzip assembler program v0.7 (5) and were used to polish the assembly sequence with SMRT Link v4.0.0.190159 (Application: Resequencing). For Illumina HiSeq 2500 sequencing, genomic DNA was fragmented to an average size of 600 bp with the DNA shearing system M220 (Covaris Inc., MA). A paired-end library was constructed with a TruSeq DNA PCR-free library prep kit (Illumina) and was size selected on an agarose gel using a Zymoclean large fragment DNA recovery kit (Zymo Research, CA). The final paired-end library was sequenced on the Illumina HiSeq 2500 sequencer with a read length of 250 bp. In the HiSeq sequencing, read quality control was performed prior to assembly by the average quality value (QV) of the reads during each cycle. Illumina HiSeq reads were mapped against the assembled PacBio contigs with BWA-MEM, and single nucleotide polymorphisms (SNPs) and indels were identified and corrected using Pilon v1.22 (6).

A summary of the genomic analysis results of the *M. gelida* MGH-2 is shown in Table 1.

**TABLE 1:** Assembly summary for genomic data of *Mrakia gelida* MGH-2

Characteristic	Data
Sampling site	Surikogi Ike, Skarvsnes ice-free area
No. of scaffolds	25
No. of reads by HiSeq 2500 instrument	6,344,609,500
Coverage (×) by HiSeq 2500 instrument	175.9
No. of reads by PacBio Sequel instrument	8,042,485,732
Coverage (×) by PacBio Sequel instrument	230.5
Total length (Mb)	34.9
GC content (%)	55.7
Scaffold *N*_50_ value (kb)	1,793
Avg length (kb)	1,395
Gen Bank accession no.	BRON00000000.1
DRA accession no.	DRA014407

Antarctica is one of the last frontiers for bioprospecting, along with deep sea and space. We believe that genomic information on *M. gelida* MGH-2 will be useful in the search for new bioactive substances and new pharmaceutical raw materials.

### Data availability.

This whole-genome shotgun project has been deposited at DDBJ/EMBL/GenBank under BioProject no. PRJDB13796 with the accession no. BRON00000000.1. The raw reads are available under the DRA accession no. DRA014407.
